# An Omics Perspective on *Candida* Infections: Toward Next-Generation Diagnosis and Therapy

**DOI:** 10.3389/fmicb.2016.00154

**Published:** 2016-02-16

**Authors:** S. P. Smeekens, F. L. van de Veerdonk, M. G. Netea

**Affiliations:** Internal Medicine, Radboud University Medical CenterNijmegen, Netherlands

**Keywords:** genome, transcriptome, proteome, metabolome, microbiome, mycobiome, mathematical modeling, antifungal host defense

## Abstract

*Candida* species can cause severe infections associated with high morbidity and mortality. Therefore, it is essential to gain more insight into the anti-fungal host defense response. The advent of *omics* technology and development of advanced systems biology tools has permitted to approach this in an unbiased and quantitative manner. This review summarizes the insights gained on anti-*Candida* immunity from genetic-, transcriptome-, proteome-, metabolome-, microbiome-, mycobiome-, and computational systems biology studies and discusses practical aspects and future perspectives.

## Introduction

*Candida albicans* is an opportunistic fungal microorganism which resides in the gastro-intestinal tract in healthy persons (Gouba and Drancourt, 2015). However, in some individuals colonization may lead to invasion and clinical disease: 75% of all woman experience vulvovaginal candidiasis at least once in there lifetime, and up to 5% of woman suffer from recurrent vulvovaginal candidiasis (Sobel, 1988). Furthermore, when the immune system is weakened, as in immunocompromised hosts (Wingard, 1979), *Candida* species can cause severe skin-, mucosal- and even systemic infections. In this respect, *Candida* is the fourth most common etiologic agent of sepsis in US hospitals, with 9.0% of all nosocomial blood stream infections being caused by *Candida* species. Despite current treatment strategies, the crude mortality rate associated with candidiasis remains very high at 39.2% (47.1% in intensive care unit patients and 27.0% in patients from other wards). For some *Candida* species (*C. krusei*) the crude mortality rate even reaches 58.7% (Wisplinghoff et al., 2004).

While the current knowledge of the host defense against *C. albicans*, mostly derived from classical immunological studies, has recently been reviewed (Netea et al., 2015), and the pathogenesis, diagnosis and treatment of invasive candidiasis has been recently described by Kullberg and Arendrup (2015), in the present review we will discuss the novel approaches to understand pathophysiology of *Candida* infections that make use of the novel *omics* technologies.

## Rationale

Despite current treatment regimens, systems fungal infections are still associated with high morbidity and mortality. In order to develop novel treatment strategies it is crucial to gain more insight into the host response against these infections. In the past, antifungal host defense research was based on hypothesis-driven *in vitro* or experimental studies. Although these “classical” studies can validate the importance of certain aspects of antifungal defense, they have a slow progress toward providing novel insights, and they are often poor indicators of the relative importance of certain pathways, both of which are crucial when looking for novel therapy targets. In this review we will focus on systems biology research that has been performed on the (human) host defense against fungal infections. Many studies have been performed on the *omics* of the fungal pathogen itself, but these lie beyond the scope of this review (see other reviews: e.g., Horn et al., 2012).

### Genomics

Relatively much research has been performed on the genetic susceptibility to *Candida* diseases. A recent review summarizes these findings; many of the genetic variants contributing to *Candida* susceptibility are located in pathogen recognition- or cytokine receptors and downstream signaling molecules (Smeekens et al., 2013c). Recently, Kumar et al. (2014) published the first GWAS on a fungal infection. The authors analyzed 118,989 single-nucleotide polymorphisms (SNPs) across 186 loci known to be associated with immune-mediated diseases in a cohort of 217 systemic candidemia patients and 11,920 controls. There was a significant association between candidemia and SNPs in *CD 58* (*OR* = 4.68), *LCE4A* (LCE 4A; *OR* = 4.25) and *TAGAP* (*OR* = 2.96) loci. The combination of two or more risk alleles even gave an increased risk for candidemia of 19.4-fold compared to no risk allele (Kumar et al., 2014). CD58, and adhesion molecule on antigen presenting cells, appeared to be involved in the inhibition of *Candida* germination. TAGAP on the other hand was needed for optimal *Candida*-induced TNF-α production, as shown by experiments performed in *Tagap^-/-^* mice.

### Transcriptomics

Barker et al. (2005) published a study on the transcriptional response of THP-1 cells after 3 h of stimulation with live *C. albicans* SC5314. *TNFA, IL8, CD83, MIP1A*, and *MIP1B* were among the genes up-regulated for at least twofold, whereas *CCR2* and *NCF2* were down-regulated. A novel finding was the up-regulation of *RGS1, RGS2, RGS16, down syndrome critical region 1, CXCL2, early growth response 3, FLT4*, and *TNF-α-induced protein 6*, as these genes were not previously known to be responsive to *C. albicans* (Sobel, 1988; Barker et al., 2005). In 2008, the authors published a similar study on vascular endothelial cells, which responded quite differently on *Candida* stimulation; genes in chemotaxis, angiogenesis and inhibition of apoptosis were mostly up-regulated in endothelial cells (Wingard, 1979; Barker et al., 2008). Also in human umbilical vein endothelial cell lines *C. albicans* induced differential expression of genes involved in apoptosis and cell death (Wisplinghoff et al., 2004; Muller et al., 2007; Li et al., 2011). Apparently the cell type under investigation has great influence on the resulting transcriptional profile after *Candida* stimulation; In human epithelial cells stimulation with *C. albicans* causes an up-regulation of genes from the NF-κB, mitogen-activated protein kinase and phosphoinositide 3 kinase/protein kinase B immune signaling pathways (Moyes et al., 2014; Netea et al., 2015), and of chemokines and adhesion molecules (Ikuta et al., 2012; Kullberg and Arendrup, 2015). On the other hand, in human granulocytes (neutrophils) *C. albicans* induces transcription of genes involved in cell-cell signaling, cell signal transduction and cell growth (Fradin et al., 2007; Horn et al., 2012).

In contrast, some stimuli induce such a strong effect that this can be observed in multiple species and cell types. In the murine macrophage cell line J774.2, *C. parapsilosis* induces transcription of genes from stress-, inflammation-, chemokine-, and cytokine pathways. The highest differential expression was seen with the *TNFRSF9* gene, which was 39-fold increased after 8 h of stimulation. Also in peritoneal macrophages from Bagg albino/c mice and in human PBMC-derived macrophages *TNFRSF9* was strongly up-regulated after *C. parapsilosis* stimulation (Kumar et al., 2014; Németh et al., 2014).

Transcriptomic studies on the pathogen side can also throw a light upon the reaction of the host immune system to the fungus. In order to do this, Fradin et al. (2005) exposed *C. albicans* to different blood fractions. *C. albicans* thrives in the absence of neutrophils. In contrast, in the presence of neutrophils *C. albicans* increases responses to overcome nitrogen- and carbohydrate starvation and genes involved in the oxidative stress response are upregulated. These results suggest that neutrophils play an essential role in the defense against *C. albicans* (Fradin et al., 2005).

Transcriptomics cannot only be used to learn new things about the host immune response, it might also be used as a diagnostic tool; in a murine infection model candidemia could accurately be classified and distinguished from *Staphylococcus aureus* infection with a sensitivity and specificity of 98 and 96%, respectively. Furthermore, by looking at the transcription profile, also infection progression could be accurately predicted; day one after infection there is increased expression of *CXCL2, CXCL13*, and *IL10R*; day two after infection there is increased expression of genes in the TNF-NF*κB*-B-cell lymphoma 2 pathway; whereas at day three and four, there is down-regulation of apoptotic genes but up-regulation of genes in Toll-like receptor pathways, inflammatory cytokines, of genes involved in NF*κB*-dependent and mitogen-activated protein kinase-dependent signaling cascades, and of genes involved in T cell activation (Zaas et al., 2010). Also in human blood, the transcriptional profile after bacterial stimulation (*S. aureus* and *Escherichia coli*) could be distinguished from that after fungal stimulation (*C. albicans* and *Aspergillus fumigatus*) using random forest classification (Dix et al., 2015). This information might be useful in sepsis, where early knowledge on the nature of the causative agent is crucial for selecting the appropriate anti-microbial therapy.

Another transcriptome study indicating the possibility to use transcriptomics as discovery tool was published in 2013. *Candida* infection could be discriminated from bacterial infection based on a 101-transcript feature set from *in vitro* experiments with human PBMC. Interestingly, in this set of 101 genes, genes from the type I interferon pathway were significantly overrepresented. This was unexpected as type I interferons had previously been associated with viral and bacterial infections. However, these findings were validated on a functional level in humans (Smeekens et al., 2013b), and also in independent mice studies (Majer et al., 2012; del Fresno et al., 2013). In a separate transcriptome experiment in humans, *IFIH1*, also part of the type I interferon signaling pathway, was implicated in the host defense against *C. albicans* hyphae. This finding was also validated on a functional level; patients with CMC express lower levels of *IFIH1*, there is a strong correlation between genetic variation in *IFIH1* and candidemia, and cells from *IFIH1* knock out mice and PBMC with different *IFIH1* genotypes have altered cytokine production upon *C. albicans* stimulation (Jaeger et al., 2015).

Tierney et al. (2012) studied the transcriptional profile of *C. albicans* and mouse dendritic cells simultaneously. Mouse Ptx3 can bind to the *C. albicans* cell wall, and thereby regulates the transcription of fungal *Hap3* target genes, this again alters the immune response to *C. albicans*, through altered expression of mouse Mta2 target genes like *IL-2* and *IL-4* (Tierney et al., 2012). Schulze et al. (2015) described a network inference tool (NetGenerator), which constructs gene regulatory networks from time series gene expression data. They re-analyzed the Tierney dataset, taking into account variances in replicated measurement data. Again, the inter-species interactions between *Ptx3*, *Hap3*, and *Mta2* were considered robust (Schulze et al., 2015). These studies emphasize the need to take into account the fact that there is an interaction between the host and pathogen.

### Proteomics

Although studies using transcriptomics are a very insightful tool, they are not able to mirror fully the landscape of active molecules inside an organism; therefore systemic assessment of proteins is an important step for understanding fungal-immune interaction. Since the introduction of proteomics, this can be done in a systematic manner. When the murine macrophage RAW 264.7 cell line was exposed to live *C. albicans* SC5314, the regulation of several proteins was affected; annexin I, LyGDI, Hspa5, tropomyosin 5, and L-plastin were increased while eukaryotic translation initiation factor 3 subunit 5, Hsp 60, Hspa9a, glucose-regulated protein 58, and Hspa8a were decreased. This indicates that several processes are affected including cytoskeletal organization, oxidative responses and protein biosynthesis and refolding (Martínez-Solano et al., 2006). In contrast, when murine macrophages are stimulated with heat-killed *C. albicans* the overall response is more anti-inflammatory, although there are some similarities with live *C. albicans* stimulation as well (Martinez-Solano et al., 2009).

Upon separately analyzing cytosol, organelle/membrane and nucleus enriched fractions from *C. albicans*-stimulated RAW 264.7 macrophages, 17 new differentially expressed proteins were identified. These proteins are involved in pro-inflammatory and oxidative responses, immune response, unfolded protein response and apoptosis (Reales-Calderón et al., 2012). Also the amount of the membrane receptor Galactin-3, a lectin receptor that recognizes b-mannans (Jouault et al., 2006), was increased upon *C. albicans* stimulation, especially in phagocytic cups (Reales-Calderón et al., 2012). This receptor had previously been implicated in the pathophysiology of infection by a transcriptome study in mouse macrophages infected with *C. albicans*, where a 3.4-fold up-regulation of galectin-3 was observed (Shin et al., 2006).

The first proteomics study in human cells stimulated with *Candida* was published in 2014. The differences in proteomes between human M1 and M2 macrophages were studied upon stimulation with *C. albicans*. The biggest differences were found in cytoskeletal rearrangement and metabolic routes; fructose-1,6-biphosphatase 1, a glycogenesis enzyme, is up-regulated in M1 macrophages. Interestingly, stimulation with *C. albicans* has been suggested to induce a polarization switch from M1 to M2, although one could argue that there is no full correlation between cellular markers and the function of the cell. This switch could be interpreted either as an attempt of the host to reduce damage caused by inflammation, or it could be a *Candida* pathogenicity mechanism by decreasing the immune response (Reales-Calderón et al., 2014).

Proteomics can also be applied to the *in vivo* situation; in human cervical-vaginal fluids almost half of the proteins are plasma components. The presence of Alb, transferrin, immunoglobulins, apolipoproteins, alpha-1-acid glycoprotein 1 and calgranulins is positively correlated with the amount of polymorphonuclear leukocytes, but there is no difference in the protein maps of asymptomatic women and women experiencing vulvovaginal candidiasis (Tang et al., 2007). In contrast, in non-neutropenic patients, invasive candidiasis could be predicted by measuring their serum antibody signature against the *C. albicans* proteins Hsp90 and Eno1 prospectively (Pitarch et al., 2014).

Proteomics can be used to investigate specific proteins. Dectin-1 is an important C-type lectin receptor in the recognition of *C. albicans*, which recognizes β-1,3- and β-1,6-glucans. In order to investigate early Dectin-1 signaling, the interactome of the intracellular receptor tail was explored using a pull-down proteomics approach in mouse CD11c^+^ cells. The SHIP-1 colocalizes with Dectin-1 during zymosan phagocytosis, and relocates to *C. albicans*-containing phagosomes. Further experiments with SHIP-1 deficient granulocyte-macrophage colony-stimulating factor-derived bone marrow cells demonstrated an important role for SHIP-1 in the regulation of reactive oxygen species production (Blanco-Menendez et al., 2015).

### Metabolomics

Metabolome studies in individuals with *Candida* infections are scarce. There is one study in which the urine metabolic profile was compared between a preterm neonate with *C. parapsilosis* infection and 13 preterm controls. *N*-glycine, D-serine, L-threonine, D-glucose, and maltose levels are higher in urine during *C. parapsilosis* infection, whereas citric acid, hexadecanoic acid and octadecanoic acid are decreased. Interestingly, therapy efficacy can be evaluated based on the metabolic profile (Dessì et al., 2014).

### Microbiome

Several studies suggest that the microbiome composition has an important impact on health status (Pflughoeft and Versalovic, 2012). The microbiome composition can be influenced by diet. In a mice study, dietary coconut oil reduced *C. albicans* colonization of the gastro-intestinal tract (Gunsalus et al., 2015). *Candida* colonization is also influenced by quantitative and qualitative aspects of the microbiome. Kennedy and Volz (1985) described that *Lactobacilli* inhibit fungal adhesion and growth by producing H_2_O_2_ and bacteriocin-like compounds. Also short-chain fatty acids from *Lactobacilli* can inhibit fungal growth (Noverr and Huffnagle, 2004). Furthermore, *Pseudomonas aeruginosa* and *Enterococcus faecalis* can inhibit *C. albicans* hyphae formation (Hogan et al., 2004). Also in a *Caenorhabditis elegans* model, *E. faecalis* inhibits *C. albicans* hyphal morphogenesis (Cruz et al., 2013). On the other hand, *C. albicans* can coaggregate with *Streptococci*, which may facilitate the colonization of oral surfaces by the yeast (Jenkinson et al., 1990).

Not only can the microbiome composition influence the extent of *Candida* colonization, it can also influence the immune response against *Candida* species (Kolwijck and van de Veerdonk, 2014; Oever and Netea, 2014; Romani et al., 2014). Mice treated with the short-chain fatty acid propionate, which also increases after the consumption of dietary fermentable fibers, have enhanced generation of macrophage and dendritic cell precursors, more dendritic cells in the lungs and reduced Th2 effector function. This demonstrates that changes in diet and metabolism can alter the immunological environment in the lungs (Trompette et al., 2014).

Also in humans the microbiome composition has been demonstrated to influence the immune response against *C. albicans;* in patients with hyper IgE syndrome and CMC, there are reduced numbers of regular skin microbiome members (e.g., *Corynebacteria*), but more Gram-negative bacteria (e.g., *Acinetobacter*), of which the latter in *in vitro* stimulation experiments with PBMC suppress the *S. aureus* and *C. albicans*-induced cytokine production (Smeekens et al., 2013a). The vaginal microbiome of patients with vulvovaginal candidiasis is highly variable, and could not be described by any single profile (Liu et al., 2013).

How the microbiome influences the immune system is not completely clear yet. One mechanism proposed by Zelante et al. (2013) is that *lactobacilli* in the gut can use tryptophan as their energy source and produce indole-3-aldehyde in the process. Indole-3-aldehyde stimulates the aryl hydrocarbon receptor, which induces IL-22 production in NKprotein46^+^NK1.1^low^ cells, that are in turn protective against *Candida* colonization at mucosal surfaces (Zelante et al., 2013).

### Mycobiome

Not only antifungal immunity is affected by microbiome composition; also fungal colonization can be influenced by the host microbiome. The fungal microbiome, also known as mycobiome, is a relatively novel research area. In terms of abundance fungi are relatively rare on human skin (Findley et al., 2013). However, when the normal balance is disturbed, e.g., in immunocompromised hosts, resident fungi can expand (Huffnagle and Noverr, 2013). Indeed, antibiotic treatment in mice causes an altered gut microbiome that coincides with outgrowth of commensal *Candida* species in the gut (Kim et al., 2014). Also genetic factors contribute to mycobiome composition; in dectin-1 deficient mice there is an altered gut mycobiome, which subsequently increases the susceptibility to experimental colitis (Iliev et al., 2012).

Oral microbiome analysis revealed that *C. albicans* was the most common fungal microorganism in healthy controls and HIV-infected participants. *Candida* colonization is negatively correlated with *Pichia* abundance. *Pichia* conditioned medium inhibits *Candida* growth and biofilms. Interestingly, in a murine oral candidiasis model *Pichia* conditioned medium lowers infection score, fungal burden, and tongue epithelial damage. This study demonstrates that findings from mycobiome data can lead to novel interesting antifungals (Zelante et al., 2014).

### Computational Systems Biology

Technical advances in the last years have increased the availability of ‘*omics*’ data, thereby augmenting the demands for mathematical modeling. As mentioned before, it is important to consider there is an ongoing interaction between cells, proteins and metabolites of pathogens and the host, which influences the outcome of infection. Dühring et al. (2015) recently gave an overview of host defense strategies and the corresponding fungal evasion mechanisms, and the computational systems biology approaches to investigate these interactions. Durmuş et al. (2015) also reviewed the available literature on the computational analysis of PHI networks, and in addition the use of web-based databases and text-mining tools in order to optimize use of currently existing data. Last year, PHI networks for *C. albicans* and *A. fumigatus* were constructed, based on protein orthology and gene function. These networks predicted Eno1, phospholipase B, Hsp70, pyruvate kinase (CDC19) as *Candida* virulence factors, which interact with the host factors CD4, Alb and amyloid beta (A4) precursor protein, Toll-like receptor 2, and epidermal growth factor receptor, respectively. These networks can serve as a framework in the analysis of host-pathogen transcriptome and proteome data (Remmele et al., 2015). It is also possible to incorporate the migration of interacting immune and *Candida* cells in three-dimensional space in a PHI network (Lehnert et al., 2015). Building a more detailed and accurate PHI model may aid timely stratification of sepsis patients based on their inflammatory status in the future.

## Discussion

Several studies have demonstrated that *Candida* infection can be discriminated from bacterial infection based on the induced transcriptome profile (Zaas et al., 2010; Smeekens et al., 2013b), suggesting that in the future transcriptomics might be used in diagnostics. However, these results should be replicated first in independent studies. This will be challenging because it is difficult to recruit large cohorts of candidemia patients. Furthermore, transcriptome studies are fairly costly and time-consuming, while rapid diagnostics are desirable. Moreover, analysis of transcriptome data is not straightforward and should be standardized between the studies (Jafari and Azuaje, 2006). Still, functional genomics studies are very useful, because they can lead to the discovery of novel therapy options. Microbiome studies have indicated several (products of) microorganisms that can inhibit *Candida* growth, like *Pichia* (Zelante et al., 2014), *Lactobacilli* (Kennedy and Volz, 1985; Noverr and Huffnagle, 2004; Zelante et al., 2013), *P. aeruginosa* and *E. faecalis* (Hogan et al., 2004). More research is necessary to utilize this knowledge in the development of novel treatment options.

There are some things to take into consideration when conducting or analyzing functional genomics studies. First of all, many studies do not demonstrate sample size calculations, and cohorts are usually small, introducing the possibility of false-positive findings and over-fitting of data to the specific cohort under investigation. Therefore, validation is an import issue. Many findings have not been validated in independent cohorts, making it difficult to assess their value. Of course, for *in vivo* studies it is difficult to gather large cohorts of patients, as invasive *Candida* infections are relatively scarce, especially from an individual medical center point of view. In the urine metabolome study there was one premature baby (32 weeks gestational age) with a fungal chest infection, who was compared to 13 premature healthy babies (mean gestational age 34.6). With only one patient it is difficult to discern the true effect on the metabolome of fungal infection from other (external) factors like in this case respiratory distress and gestational age (Dessì et al., 2014).

Next to adequate samples size and validation in independent cohorts it is also important to keep in mind the functional validation of novel findings. Employing systems biology will always lead to a top list of genes, proteins, metabolites, or microorganisms associated with a certain phenotype like *Candida* infection. However, this does not mean that these associations are also relevant. Furthermore, it is difficult to interpret novel, unexpected findings, without knowing their functional consequence(s). Therefore results need to be validated on a functional level. For example, Zelante et al. (2014) demonstrated that *Candida* colonization was negatively correlated with *Pichia* abundance in HIV patients, which is a purely statistical finding. They strengthened this finding by demonstrating that *Pichia* conditioned medium inhibits *Candida* growth and biofilm formation. More importantly, *Pichia* conditioned medium ameliorated a murine oral candidiasis model (Zelante et al., 2014). Furthermore, Kumar et al. (2014) demonstrated in a GWAS study that SNPs in *CD58*, *LCE4A*, and *TAGAP* were significantly associated with candidemia. Two of the genes were validated on a functional level; in siRNA experiments *CD58* appeared to be involved in the inhibition of *Candida* germination. *Tagap^-/-^* mice had reduced *Candida*-induced TNF-α production (Kumar et al., 2014).

**FIGURE 1:**
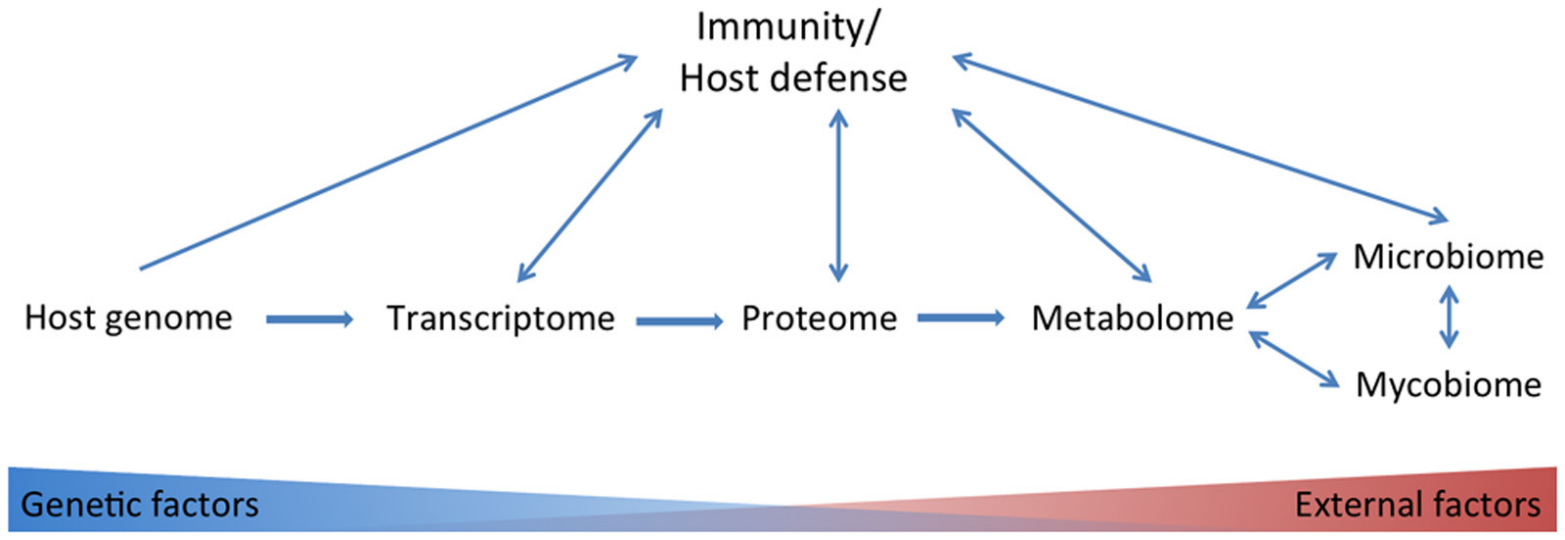
**Overview of omics tools used in studies on the anti-*Candida* host defense.** Host genetic-, transcriptome-, proteome-, metabolome-, microbiome-, and mycobiome data have been linked to immunity/host defense. Host genetic factors play to a lesser extent a role in consecutively transcriptome, proteome, metabolome, and microbiome and mycobiome data.

There is no golden standard in systems biology approaches. The correlation between different systems biology approaches is not 100%; in mice it has been reported that for only about half of the genes tested there was a correlation between transcripts and proteins, with an average correlation of 0.27 (Ghazalpour et al., 2011). Compared to the proteome the metabolome is even less subject to genetic influences but more to environmental factors (**Figure 1**). In a study with healthy male volunteers, up to 12% of the observed variance in metabolite concentrations could be explained by genetic variation in common SNPs (Gieger et al., 2008). Although the transcriptome, proteome and metabolome are less subject to genetic influences, they are often more closely related to phenotypic traits compared to genome data (Homuth et al., 2012). In contrast, clinical traits, like adiposity, were more strongly correlated to transcript levels than to protein levels in a study with mice (Ghazalpour et al., 2011).

Also the models and experimental setups that are chosen have great influence on systems biology approaches. As discussed above, *C. albicans* induces the transcription of different principal pathways in human monocyte-like cells (Barker et al., 2005), vascular endothelial cells (Muller et al., 2007; Barker et al., 2008; Li et al., 2011), human epithelial cells (Ikuta et al., 2012; Moyes et al., 2014) and human granulocytes (neutrophils; Fradin et al., 2007). The fungal species is of importance as well, and *TNFRSF9* transcription has so far only been shown to be up-regulated after *C. parapsilosis* stimulation (Németh et al., 2014). Most studies have been performed with *C. albicans*, while other fungal species, like *C. tropicalis*, *C. glabrata*, and *C. krusei*, are also medically relevant (Kullberg and Arendrup, 2015). These should also be the focus of future studies. Another aspect is fungal strain and morphology; many studies use heat-killed fungi, while these are fundamentally different from live fungi. In PBMC cultures live *C. albicans* can actively suppress IL-17 production (Cheng et al., 2010), and important cytokine in the anti-*Candida* host defense (van de Veerdonk et al., 2009). So although using live fungal pathogens more closely resembles an *in vivo* infection, it is more difficult to delineate host defense responses from fungal influences.

In the past few years, functional genomics studies have led to interesting novel discoveries regarding the anti-*Candida* host defense (**Table 1**), making them a valuable addition to classical immunological studies.

**Table 1:** Research highlights from functional genomics studies on the host immune response against *Candida albicans*

Tool	Model	Main findings	Reference
Genomics	GWAS with human candidemia patients	SNPs in CD58, LCE4A, TAGAP increase risk for candidemia	Kumar et al., 2014
Transcriptomics	*In vitro* studies with human monocyte cell line or mouse dendritic cells	Differential expression of specific genes: RGS1, RGS2, RGS16, *down syndrome critical region 1*, CXCL2, *early growth response 3, FLT4*, *TNF-α-induced protein 6, Ptx3*, and *Mta2*	Barker et al., 2005; Tierney et al., 2012; Schulze et al., 2015
	Endothelial cells stimulated with *C. albicans*	Differential regulation of certain pathways: Type I interferons; chemotaxis, angiogenesis, apoptosis, cell death, NF-κB- MAPK- and PI3K/Akt signaling; cell–cell signaling, cell signal transduction and cell growth, chemokines and adhesion molecules	Wisplinghoff et al., 2004; Fradin et al., 2007; Muller et al., 2007; Barker et al., 2008; Li et al., 2011; Horn et al., 2012; Ikuta et al., 2012; Smeekens et al., 2013b; Moyes et al., 2014; Kullberg and Arendrup, 2015; Netea et al., 2015
	Murine candidemia model, *in vitro* studies with human blood	Transcriptomics can be used as a diagnostic tool to discriminate fungal from bacterial infection	Zaas et al., 2010; Dix et al., 2015
Proteomics	Serum from non-neutropenic patients	Proteomics can be useful in prognosis: the serum antibody signature against the *C. albicans* proteins Hsp90 and Eno1 is predictive of invasive candidiasis	Pitarch et al., 2014
	*In vitro* studies with human and murine macrophages	Differential regulation of certain processes: cytoskeletal organization, oxidative responses, protein biosynthesis and refolding, pro-inflammatory responses, immune response, unfolded protein response and apoptosis and metabolism	Martínez-Solano et al., 2006; Reales-Calderón et al., 2012, 2014; Blanco-Menendez et al., 2015
Metabolomics	Urine metabolome in *C. parapsilosis* neonate	Increased levels of *N*-glycine, D-serine, L-threonine, D-glucose and maltose levels. Decreased levels of citric acid, hexadecanoic acid and octadecanoic acid	Dessì et al., 2014
Microbiome	*In vitro* experiments	Microbiome composition can influence *Candida* colonization	Hogan et al., 2004; Noverr and Huffnagle, 2004
	Microbiome studies in patients with skin/mucosal *C. albicans* infections, mice studies	Microbiome composition influences the host immune response against *C. albicans*	Zelante et al., 2013; Smeekens et al., 2013a; Trompette et al., 2014
Mycobiome	Oral microbiome analysis in HIV patients, *in vitro* studies and a murine oral candidiasis model	*Pichia* inhibits *C. albicans*	Zelante et al., 2014
Computational systems biology	Construction of PHI network	The host factors CD4, Alb and amyloid beta (A4) precursor protein, Toll-like receptor 2, epidermal growth factor receptor are predicted to interact with *C. albicans* virulence factors	Remmele et al., 2015

## Author Contributions

All authors listed, have made substantial, direct and intellectual contribution to the work, and approved it for publication.

## Conflict of Interest Statement

The authors declare that the research was conducted in the absence of any commercial or financial relationships that could be construed as a potential conflict of interest.

## Abbreviations

Alb: albumin
CD: cluster of differentiation
CMC: chronic mucocutaneous candidiasis
CXCL: chemokine (C-X-C motif) ligand
Eno: enolase
GWAS: genome wide association study
Hap: hook-associated protein
Hsp: heat shock protease protein
IFIH: interferon induced with helicase c domain
IL: interleukin
LCE: late cornified envelope
Mta: metastasis-associated protein
NF: nuclear factor
OR: odds ratio
PHI: pathogen-host interaction
Ptx: pentraxin
MIP: macrophage inflammatory protein
PBMC: peripheral blood mononuclear Cells
RGS: regulator of G-protein signaling
SHIP: SH2 domain containing inositol phosphatase
SNP: single nucleotide polymorphism
TAGAP: T cell activation RHO-GTPase-activating protein
TNF: tumor necrosis factor
TNFRSF: tumor necrosis factor super family
